# Mutations Selected After Exposure to Bacteriocin Lcn972 Activate a Bce-Like Bacitracin Resistance Module in *Lactococcus lactis*

**DOI:** 10.3389/fmicb.2020.01805

**Published:** 2020-08-13

**Authors:** Ana Belén Campelo, María Jesús López-González, Susana Escobedo, Thomas Janzen, Ana Rute Neves, Ana Rodríguez, Beatriz Martínez

**Affiliations:** ^1^DairySafe group, Department of Technology and Biotechnology of Dairy Products, Instituto de Productos Lácteos de Asturias (IPLA), Consejo Superior de Investigaciones Científicas (CSIC), Villaviciosa, Spain; ^2^Instituto de Investigación Sanitaria del Principado de Asturias (ISPA), Oviedo, Spain; ^3^Chr. Hansen A/S, Hørsholm, Denmark

**Keywords:** cell wall, bacteriocin, antimicrobial peptides, ABC transporter, resistance, *Lactococcus lactis*

## Abstract

Resistance against antimicrobial peptides (AMPs) is often mediated by detoxification modules that rely on sensing the AMP through a BceAB-like ATP-binding cassette (ABC) transporter that subsequently activates a cognate two-component system (TCS) to mount the cell response. Here, the *Lactococcus lactis* ABC transporter YsaDCB is shown to constitute, together with TCS-G, a detoxification module that protects *L. lactis* against bacitracin and the bacteriocin Lcn972, both AMPs that inhibit cell wall biosynthesis. Initially, increased expression of *ysaDCB* was detected by RT-qPCR in three *L. lactis* resistant to Lcn972, two of which were also resistant to bacitracin. These mutants shared, among others, single-point mutations in *ysaB* coding for the putative Bce-like permease. These results led us to investigate the function of YsaDCB ABC-transporter and study the impact of these mutations. Expression *in trans* of *ysaDCB* in *L. lactis* NZ9000, a strain that lacks a functional detoxification module, enhanced resistance to both AMPs, demonstrating its role as a resistance factor in *L. lactis*. When the three different *ysaB* alleles from the mutants were expressed, all of them outperformed the wild-type transporter in resistance against Lcn972 but not against bacitracin, suggesting a distinct mode of protection against each AMP. Moreover, P*_ysaD_* promoter fusions, designed to measure the activation of the detoxification module, revealed that the *ysaB* mutations unlock transcriptional control by TCS-G, resulting in constitutive expression of the *ysaDCB* operon. Finally, deletion of *ysaD* was also performed to get an insight into the function of this gene. *ysaD* encodes a secreted peptide and is part of the *ysaDCB* operon. YsaD appears to modulate signal relay between the ABC transporter and TCS-G, based on the different response of the P*_ysaD_* promoter fusions when it is not present. Altogether, the results underscore the unique features of this lactococcal detoxification module that warrant further research to advance in our overall understanding of these important resistance factors in bacteria.

## Introduction

ATP-binding cassette (ABC) transporters are bacterial efflux pumps that mediate transport across the membrane at the expenses of the energy liberated by the hydrolysis of ATP (Du et al., 2018). They are constituted by a transmembrane domain (TMD) with a substrate-binding pocket and the nucleotide-binding domain (NBD) that alternate between two structural conformations, inward-open and outward-open, to translocate substrates. ABC transporters are functionally diverse and participate in bacterial virulence, quorum sensing, nutrient uptake, or export of toxic molecules. In particular, ABC transporters are regarded as the fastest-acting and very effective resistance mechanisms to many antimicrobials (Du et al., 2018). Moreover, their role as sensors and information processors in signal relay is being increasingly recognized (Piepenbreier et al., 2017).

In Firmicutes, some ABC transporters confer resistance to antimicrobial peptides (AMPs) including peptide antibiotics (e.g., bacitracin), host antimicrobial peptides (e.g., LL-37 and hBD3 defensins), and bacteriocins (e.g., nisin). Based on their domain architecture and phylogenetic relationships, five groups have been established (Gebhard, 2012). Two of them are required for the export of newly synthesized AMPs (SunT and NisT type) and three are involved in resistance (LanFEG, BceAB, and BcrAB type). BceAB-type transporters are characterized by the NBD protein BceA and the permease BceB with 10 transmembrane helices (TMH) and a large extracellular loop (EC_L_) between TMH-VII and VIII. Many BceAB-type transporters are genetically and functionally linked to two component systems (TCS), involved in signal transduction and gene regulation. Actually, the Bce-type permeases and the histidine kinases of the cognate TCS have coevolved toward functional AMP detoxification modules (Dintner et al., 2011). Moreover, the histidine kinases of these TCSs are intramembrane-sensing kinases that completely rely on the ABC transporter to sense the AMP (Bernard et al., 2007). One of the best-characterized detoxification modules is BceRS-BceAB involved in resistance of *Bacillus subtilis* to bacitracin. The BceAB transporter actively participates in bacitracin resistance, likely by freeing its target from the antibiotic grip (Kobras et al., 2020), and interacts directly with the histidine kinase BceS to form a sensory complex that controls the expression of *bceAB* (Dintner et al., 2014). This detoxification module responds to a novel signaling mechanism, so-called flux sensing, whereby monitoring the activity of the individual ABC transporters is the cue to activating the cognate TCS and triggering the cell response (Fritz et al., 2015).

The role of BceAB-type transporters in sensing, together with their role in detoxification, has prompted their classification into three main functional groups proposed by Revilla-Guarinos et al. (2014): (i) those with a dual function involved in sensing and resistance (e.g., BceAB, ABC09 of *Lactobacillus casei*); (ii) sensing transporters that trigger the response but are unable to confer resistance on their own (e.g., VraFG that activates the GraXSR TCS in *Staphylococcus aureus*); and (iii) standalone detoxification pumps which are often regulated by a nongenetically linked TCS (e.g., VraDEH of *S. aureus*).

Additional or accessory components within the ABC/TCS resistance modules may also be present and are thought to be involved in fine tuning both signaling and transport activities. The cytosolic protein GraX from the TCS GraXRS of *S. aureus* has been shown to interact with the histidine kinase GraS, and it is required for GraR-gene activation (Falord et al., 2012). Likewise, *vraH*, located downstream in the *vraDEH* operon, encodes a small membrane protein, which is required for high-resistance to daptomycin and gallidermin (Popella et al., 2016). Other protecting mechanisms may also cluster with ABC/TCS detoxification modules. In *Streptococcus agalactiae*, the module NsrFP/NsrKR involved in nisin resistance is complemented with the nisin resistance protein Nsr, a nisin-degrading protease (Khosa et al., 2013; Reiners et al., 2017).

In a previous work, we have carried out adaptive evolution experiments with several *Lactococcus lactis* strains, which are used worldwide as starters in milk fermentation (López-González et al., 2018). These strains were grown under the selective pressure exerted by the bacteriocin Lcn972, a class IId bacteriocin that binds to lipid II and inhibits cell wall biosynthesis, triggering the cell envelope stress response in *Lactococcus* (Martínez et al., 2007, 2008). The preliminary insight into the genome of these mutants resistant to Lcn972 revealed that, among others, non-synonymous mutations often occurred in a putative Bce-like detoxification module, comprised by an ABC transporter (YsaDCB) and an adjacent TCS (TCS-G) (López-González et al., 2018). Precisely, three of these mutants shared mutations in *ysaB*, coding for a BceB-like permease and two of them were also resistant to bacitracin. These observations prompted us to hypothesize that the YsaDCB ABC transporter might be part of a Bce-like resistance module in *L. lactis*. Therefore, we set out to determine the role of YsaDCB in resistance to bacitracin and Lcn972 and to investigate the consequences of the *ysaB* mutations. The results show that *L. lactis* YsaDCB is involved in both sensing and resistance to AMPs and that those mutations in *ysaB* may impair either function. Moreover, YsaDCB is shown to protect against bacitracin and Lcn972 by a distinct mode of action. Finally, the secreted peptide YsaD is proposed to modulate signal transduction between the ABC transporter and the histidine kinase of TCS-G.

## Materials and Methods

### Bacterial Strains and Growth Conditions

*L. lactis* strains used in this work and their main characteristics are listed in Table 1. The wild-type (WT) *L. lactis* L81 and L62 and their Lcn972R mutants L81-D1, L81-E2, and L62-G9 were grown at 30°C in M17 with lactose at 0.5% (LM17). For the plasmid-free laboratory strains *L. lactis* IL1403 and *L. lactis* NZ9000, glucose at 0.5% was used (GM17). Antibiotics chloramphenicol (Cm), erythromycin (Em), and tetracycline (Tet) were added at 5 μg/ml depending on the plasmid (Table 1) and the same concentrations were used when two plasmids were present in the same lactococcal cell.

**Table 1:** Bacterial strains and plasmids used in this study.

Strain	Properties*^a^*	Reference
*L. lactis* **subsp.** *lactis*		
L81	WT strain, commercial starter culture	López-González et al. (2018)
L81-D1	Lcn972R from L81, *ysaB* mutation F_577_S, P_605_T	
L81-E2	Lcn972R from L81, *ysaB* mutation F_577_V	
L62	WT strain, commercial starter culture	
L62-G9	Lcn972R from L62, *ysaB* mutation I_594_F	
IL1403	Laboratory strain, plasmid free	Bolotin et al. (2001)
*L. lactis* **subsp.** *cremoris*		
NZ9000	Host for nisin-inducible gene expression; *ysaB* pseudogene	Kuipers et al. (1998)
**Plasmid**		
pUK200	Nisin-inducible expression vector. Cm^R^	Wegmann et al. (1999)
pRCR	Promoter-probe vector, *mrfp* (mCherry). Cm^R^	Mohedano et al. (2015)
pPTPL	Promoter-probe vector, *lacZ*. Tet^R^	Burgess et al. (2004)
pILG	Based on pIL252. Em^R^	Campelo et al. (2010)
pDCB_n	*ysaDCB* genes in pUK200, “n” stands for strain origin	This work
pCB_n	*ysaCB* genes in pUK200, “n” stands for strain origin	This work
pP*_ysaD_*::*lacZ*	*ysaD_IL_* promoter fused to *lacZ* in pPTPL	This work
pRCR_P*_ysaD_*::*mrfp*	*ysaD_IL_* promoter fused to *mrfp* in pRCR	This work
pIL_P*_ysaD_*::*mrfp*	*ysaD_IL_* promoter fused to *mrfp* in pILG	This work

a WT, wild-type; Cm, chloramphenicol; Tet, tetracycline; Em, erythromycin. L81-D1, L81-E2, and L62-G9 carry several mutations (López-González et al., 2018), but only those in ysaB are specified.

### Antimicrobial Susceptibility Tests

Minimum inhibitory concentrations (MICs) were determined by the broth dilution method in microtiter plates as previously described (López-González et al., 2018). Two-fold dilutions of the antimicrobials to be tested (bacitracin, nisin, and vancomycin) were done in GM17 or LM17 broth depending on the lactococcal strain. Plates were inoculated with exponentially growing cells adjusted to an optical density at 600 nm (OD_600_) of 0.05, and then further diluted 1/100. Susceptibility of the *L. lactis* NZ9000 clones carrying the expression plasmids pDCB_n and pCB_n (Table 1) was scored according to the inhibitory concentration able to inhibit growth by 50% (IC_50_). IC_50_ was determined essentially as described by Reiners et al. (2017). IC_50_ plates were inoculated with overnight cultures adjusted to an OD_600_ of 0.1. For gene expression, 1 ng/ml of nisin was routinely added to both pre-cultures and IC_50_ determinations.

### RNA Extraction and Analysis

Three independent cultures of *L. lactis* L81, *L. lactis* L62, and their Lcn972R mutants (Table 1) were grown on LM17 at 30°C until they reached an OD_600_ of 1.0, when RNAprotect Bacteria Reagent (Qiagen, Germany) was added. Total RNA was extracted using the Illustra RNAspin Mini Kit (GE Healthcare, UK) and treated with Turbo DNAse (Ambion) and SUPERase RNase Inhibitor (Ambion). RNA quality was checked by agarose gel electrophoresis, and its concentration was determined by absorbance at 260 nm in an Epoch microplate spectrophotometer (BioTek). One microgram of each RNA sample was used to generate cDNA with the iScript cDNA Synthesis Kit (Bio-Rad Laboratories, Hercules, CA).

RT-qPCR was performed in a 7500 Fast Real-Time PCR System (Applied Biosystems, Warrington, UK). Primers used for RT-qPCR are listed in Supplementary Table 1 and were supplied by Macrogen (Korea). Amplification was carried out in 25 μl containing 2.5 μl of a 1:25 dilution of cDNA, 1× Power SYBR Green (Applied Biosystems), and each primer at a concentration of 0.2 μM. Each cDNA amplification was repeated in duplicate. After incubation at 95°C for 10 min, amplification proceeded with 40 cycles of 95°C for 15 s and 60°C for 1 min. Fold changes were calculated following the 2^−Δ∆Ct^ method (Livak and Schmittgen, 2001), and the reference gene was the elongation factor Tu *tuf*.

PCR reactions to identify transcription units were performed in a MyCycler Thermal Cycler (Bio-Rad Laboratories). Primers are listed in Supplementary Table 1. PCR reactions were carried out in a final volume of 25 μl containing 1 μl of cDNA, 1× Taq DNA Polymerase Master Mix Red (Ampliqon, Denmark) and each primer at a concentration of 0.2 μM. PCR amplification conditions were as follows: 1 cycle at 95°C for 4 min followed by 30 cycles at 95°C for 30 s, 50°C for 30 s, and 72°C for 30 s and a final cycle at 72°C for 7 min. PCR controls were carried with either RNA or genomic DNA. PCR products were analyzed by 2% agarose gel electrophoresis.

### pDCB and pCB Expression Plasmids

All the primers required for plasmid construction are listed in Supplementary Table 1. Restriction enzymes and T4 ligase were supplied by Eurofins Genomics (Germany) and Fisher Scientific (Spain), respectively, and used according to the manufacturer’s instructions. Commercial kits were used for purification of chromosomal DNA (GenElute Bacterial Genomic DNA Kit, Sigma, Spain), plasmids (High Pure Plasmid Isolation Kit, Roche, Germany), and DNA fragments using Illustra GFX PCR DNA and Gel Band Purification Kit (GE Healthcare, UK). The expression plasmids were based on pUK200, placing the *ysaDCB* and *ysaCB* genes under the control of the inducible nisin promoter to yield the plasmids pDCB_n and pCB_n (Table 1), tagged after the source of the *ysa* genes as pDCB_IL (IL1403), pDCB_L81, pDCB_D1, pDCB_E2, pDCB_G9, pCB_IL, and pCB_L81. The genes were amplified by PCR with Pwo SuperYield polymerase (Roche, Spain) using as template genomic DNA from *L. lactis* strains IL1403, L81, D1, E2, and G9 and annealing T of 58°C. PCR products were digested with RcaI and BamHI in the case of the pDCB_n plasmids and with NcoI and BamHI for the pCB_n plasmids. After digestion, the DNA fragments were ligated into pUK200 cut with NcoI and BamHI. The resulting plasmids were established into *L. lactis* NZ9000 by electroporation and checked by DNA sequencing (Macrogen, Spain) to confirm proper cloning.

### P*_ysaD_* Reporter Plasmids

The β-galactosidase reporter plasmid pP*_ysaD_*::*lacZ* was based on pPTPL (Table 1). The promoter P*_ysaD_* was amplified by PCR using Pwo SuperYield polymerase (Roche, Spain) and the primers described in Supplementary Table 1. As template, genomic DNA from *L. lactis* IL1403 was used since the upstream *ysaD* DNA sequence was identical in *L. lactis* IL1403, L81, and L62. The PCR product and the vector were digested by EcoRI and XbaI, ligated, and electroporated into *L. lactis* NZ9000. Two fluorescent reporter plasmids harboring the *mrfp* gene coding for mCherry were constructed (Table 1). pRCR_P*_ysaD_*::*mrfp* was based on pRCR (Table 1). The PCR-amplified P*_ysaD_* promoter was digested with XbaI only to leave the 5' end blunt and ligated into SmaI-XbaI-digested pRCR. *Escherichia coli* DH10B (Invitrogen) was used as an intermediate host for cloning. The plasmid was introduced into *L. lactis* strains IL1403 and NZ9000 and the lactose positive *L. lactis* L81, L81-D1, and L81-E2 by electroporation. The BglII-SacI DNA fragment encompassing the P*_ysaD_*::*mrpf* fusion from pRCR_P*_ysaD_*::*mrfp* was excised and cloned into pILG, digested with the same restriction enzymes to yield pIL_P*_ysaD_*::*mrfp* (Table 1). This plasmid carries an erythromycin resistance marker, which made possible to maintain the reporter plasmid together with pDCB_n and pCB_n expression plasmids (Cm^R^) in the same cell. Correct cloning was confirmed by DNA sequencing (Macrogen, Spain).

### Activity of the P*_ysaD_* Promoter With the Reporter Plasmid pP*_ysaD_*::*lacZ*

Fresh overnight cultures of *L. lactis* NZ9000 clones carrying the different expression plasmids pDCB_n and pCB_n and the reporter plasmid pP*_ysaD_*::*lacZ* were diluted to OD_600_ of 0.05 in GM17/Cm/Tet and nisin at 1 ng/ml to ensure expression of the *ysa* genes. When the clones reached an OD_600_ of 0.2–0.3 (approximately 3 h at 30°C), 2 ml samples were taken and bacitracin was added at 5 and 1 μg/ml and incubation proceeded for 30 min. Cultures without bacitracin were taken as baseline controls. Cells were collected by centrifugation, and the β-galactosidase reaction was carried out at 30°C for 10 min as described elsewhere (Martínez et al., 2007). β-Galactosidase activity assays were carried out, at least, with three independent cultures.

### Activity of the P*_ysaD_* Promoter With the Reporter Plasmid pIL_P*_ysaD_*::*mrfp*

*L. lactis* NZ9000 pDCB_n and pCB_n and the reporter plasmid pIL_P*_ysaD_*::*mrfp* were inoculated in GM17/Cm/Em and 1 ng/ml nisin and grown as described for pP*_ysaD_*::*LacZ*. After induction with bacitracin (1 and 5 μg/ml) or Lcn972 (80, 40, and 20 AU/ml), cells (1 ml) were washed with saline phosphate buffer (PBS), pH 7.3, and collected in half of the initial volume. Cell suspensions were kept in the dark for 3 h for mCherry maturation (Garay-Novillo et al., 2019). Fluorescence (F) of 20-μl aliquots was quantified in a 7500 Fast Real-Time PCR System (Applied Biosystems, Warrington, UK) using the built-in presence/absence protocol for detecting ROX, a fluorophore with similar excitation (Ex. 580 nm) and emission (Em. 605 nm) wavelengths as mCherry (Ex. 587 nm, Em. 612 nm). After blank correction (PBS background), raw fluorescence data was normalized by dividing F by the OD_600_ of the cell suspensions (200 μl) measured in a Benchmark Plus microplate spectrophotometer (Bio-Rad).

### Statistical Analysis

When indicated, differences were assessed by one-tailed *t*-test as implemented in Microsoft Excel 2010 (2010 Microsoft Corporation) and *p* < 0.05 was considered to be significant.

## Results

### Resistance of Lcn972R *L. lactis* Mutants to AMPs Other Than Lcn972

In our previous work, three Lcn972 resistant mutants (Lcn972R) L81-D1, L81-E2, and L62-G9 (Table 1) were isolated after continuous exposure of the wild-type (WT) strains *L. lactis* subsp. *lactis* L81 and *L. lactis* subsp. *lactis* L62 to subinhibitory concentrations of the cell wall-active bacteriocin Lcn972. The preliminary phenotypic characterization already suggested that the two mutants L81-E2 and L62-G9 displayed cross-resistance to bacitracin, while L81-D1 was more susceptible than its parent *L. lactis* L81 (López-González et al., 2018). Standard MIC determinations confirmed that bacitracin MICs for L81-E2 and L62-G9 increased by 8‐ and 4-fold, respectively, whereas the MIC for *L. lactis* L81-D1 was reduced 2-fold (Table 2). MICs of other antimicrobials such as nisin and vancomycin also increased 2‐ or 4-fold for some Lcn972R mutants (Table 2) in line with our previous results (López-González et al., 2018).

**Table 2:** Minimum inhibitory concentrations (MICs) of several antimicrobial peptides for *L. lactis* strains.

*L. lactis*	MIC			
	Lcn972 (AU/ml)**^a^**	Bacitracin (μg/ml)	Vancomycin (μg/ml)	Nisin (μg/ml)**^b^**
L81 (WT)	10	8	0.5	1
L81-D1	80	4	0.5	2
L81-E2	320	64	0.5	4
L62 (WT)	10	8	0.5	2
L62-G9	80	32	1	4
IL1403	40	0.125	0.5	ND
NZ9000	20	0.5	0.5	ND

a Taken from López-González et al. (2018).

b ND, not determined.

### Presence of a BceAB-Like Detoxification Module in *L. lactis*

**Figure 1:**
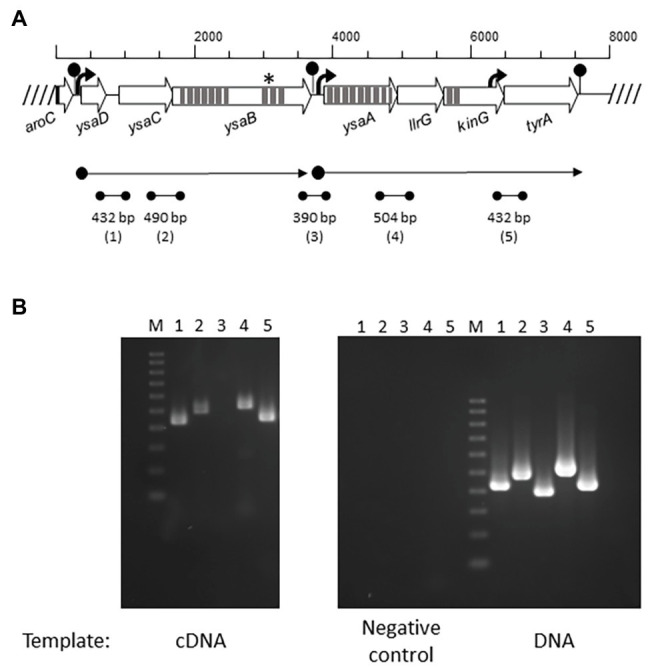
Operon organization of the Ysa TCS-G module in *Lactococcus lactis*. **(A)** Overview of the *ysa* and flanking genes (open arrows). Promoters are depicted as black arrows and terminators as lollypops. Predicted transmembrane helices are shown as dark gray strips. Localization of *ysaB* mutations is marked with an asterisk (^*^). The two predicted mRNAs are displayed by dot-end arrows. Intergenic regions (1–5) amplified by RT-PCR are shown by dot-end lines. **(B)** RT-PCR amplified bands (1–5) using as template cDNA, RNA (negative control), and genomic DNA (positive control) from *L. lactis* L81.

Three Lcn972R mutants L81-D1, L81-E2, and L62-G9 carried, among others, non-synonymous mutations in *ysaB*, a gene coding for the permease of a putative ABC transporter (López-González et al., 2018). Inspection of the genome of the WT strains L81 and L62 (European Nucleotide Archive under accession number ENA: PRJNA 492214) and the laboratory strain *L. lactis* subsp. *lactis* IL1403 (GenBank AE005176.1) revealed that *ysaB* is flanked by *ysaC* and genes encoding the TCS-G (Figure 1A). YsaC contains the ATP-binding domain and YsaB shares the features of BceB-like permeases with 10 transmembrane helices (TMHs) and an extracellular loop (233 aa), according to TMpred^1^ and Psort^2^ predictions. All the *ysaB* mutations detected in the Lcn972R strains mapped in the C-terminus. Three mutations (F_577_V, F_577_S, and I_594_F) were found in the predicted inside loop between TMH VIII and IX. The additional mutation P_605_T detected in *L. lactis* L81-D1 was positioned within TMH IX. The genes encoding the TCS-G with the response regulator LlrG and the intramembrane histidine kinase KinG are located downstream of *ysaB* (Figure 1A). This genetic arrangement is similar to that found for other BceAB-like ABC transporters which are genetically linked to regulatory elements such as TCSs and involved in resistance to AMPs (Dintner et al., 2011).

Additional genes (*ysaD* and *ysaA*) were also identified (Figure 1A). Homology and conserved domain searches identified YsaD (119 aa) as a member of TIGR01655 (uncharacterized protein YxeA), a family of small uncharacterized secreted proteins which are found exclusively in Gram-positive bacteria. YsaA (354 aa) is predicted to be an integral membrane protein with nine TMHs. It contains a VanZ domain present in glycopeptide antibiotic resistance proteins (pfam04892), denoting a possible role in resistance to AMPs.

The module YsaDCB/TCS-G is present in 41 out of the 192 complete *L. lactis* genomes deposited at NCBI (as of June 2020) according to BLASTN^3^ searches, and it seems highly conserved (over 80% identity at the nucleotide level) within the two *L. lactis* subspecies *cremoris* and *lactis*. It is worth mentioning that, in the laboratory workhorse strain *L. lactis* MG1363 and its derivative *L. lactis* NZ9000, the ABC transporter appears to be nonfunctional due to a stop codon at position 310 that created a truncated YsaB permease of 103 residues. Otherwise, its TCS-G is complete. A detailed comparison at the protein level and accession numbers is included in Supplementary Table 2.

### The Putative Detoxification Module YsaDCB/TCS-G in *L. lactis* is Transcribed in two Polycistronic mRNAs

The genes of the putative Ysa-TCS-G module are transcribed in two polycistronic mRNAs as determined by RT-PCR using primers annealing within the gene ends (Figure 1B). Based on the amplification results, two mRNAs *ysaDCB* and *ysaA-llrG-kinG-tyrA* are synthesized. Consistent with this, two putative promoters could be identified upstream of *ysaD* (P*ysaD*) and *ysaA* (P*ysaA*), as well as three rho-independent terminators downstream of *aroC*, *ysaB*, and *tyrA* (Figure 1B). A positive PCR amplification of the intergenic region *kinG*-*tyrA* was detected, which could be due to the presence of a putative promoter at the 3' end of *kinG*, upstream of the forward primer used in RT-PCR (Figure 1B). The flanking genes *tyrA* and *aroC* coding for prephenate dehydrogenase and chorismate synthase, respectively, are involved in the biosynthesis of aromatic amino acids. Hence, it is unlikely that they participate in the transport and/or sensing functions of the YsaDCB/TCS-G module.

### *ysaDCB* but Not TCS-G Genes are Highly Expressed in Lcn972R *L. lactis* Mutants

**Figure 2:**
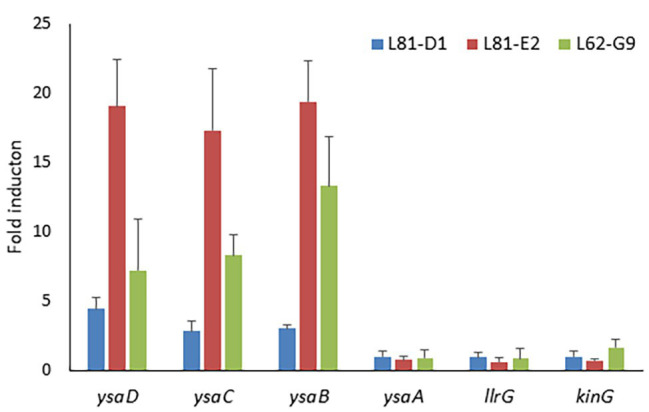
Relative expression of Ysa and TCS-G genes in the *L. lactis* Lcn972R mutants determined by RT-qPCR. Relative gene expression was determined by the 2^−ΔΔCt^ method using the WT strains *L. lactis* L81 (for L81-D1 and L81-E2) and *L. lactis* L62 for L62-G9 as reference. Average of three biological replicates and SD (error bars) are shown.

Bearing in mind the role of BceAB-like transporters in resistance to AMPs, the expression levels of *ysaDCB*, *ysaA*, *llrG*, and *kinG* were determined in exponentially growing cultures of the *L. lactis* Lcn972R mutants by RT-qPCR. The expression of the TCS-G genes was not altered in any of the mutants. On the contrary, *ysaDCB* were upregulated in the Lcn972R mutants albeit to a different extent. Highest expression occurred in *L. lactis* L81-E2 (close to 20-fold), followed by *L. lactis* L62-G9 (up to 10-fold; Figure 2). In *L. lactis* L81-D1, which carries two *ysaB* mutations (F_577_S and P_605_T) and is sensitive to bacitracin, the expression of *ysaDCB* was lower but still up to 5-fold compared to the WT *L. lactis* L81 (Figure 2). Closer inspection and resequencing of the PCR-amplified P*_ysaD_* promoter in all these strains did not show any anomalies, which could explain the higher expression levels of the *ysaDCB* operon in the Lcn972R mutants.

Further confirmation of the increased expression of the *ysaDCB* genes in the Lcn972R *L. lactis* mutants was gathered by monitoring the functional expression of the fluorescent mCherry protein gene *mrfp* placed under the control of the P_ysaD_ promoter in the reporter plasmid pRCR_P*_ysaD_*::*mrfp*. While hardly any fluorescence could be measured in both exponential and stationary phase cultures of *L. lactis* L81, fluorescence was 30-fold and 10-fold higher in *L. lactis* L81_E2 and *L. lactis* L81_D1 (Supplementary Figure S1A).

Of note, the expression levels of *ysaDCB* mirrored resistance to Lcn972 but not to bacitracin (Table 2). Despite their increased expression in *L. lactis* L81-D1, this mutant was more sensitive to bacitracin than the WT. Moreover, different susceptibility to bacitracin was also noticed within the laboratory strains *L. lactis* IL1403, with a complete YsaDCB transporter, and *L. lactis* NZ9000 that lacks a functional YsaB permease, being 4-fold more resistant to bacitracin than IL1403. These observations suggest that bacitracin resistance mechanisms other than YsaDCB are functional in *L. lactis*.

### Mutated Versions of *ysaB* Provide Distinct Resistance Levels to Bacitracin and Lcn972

**Figure 3:**
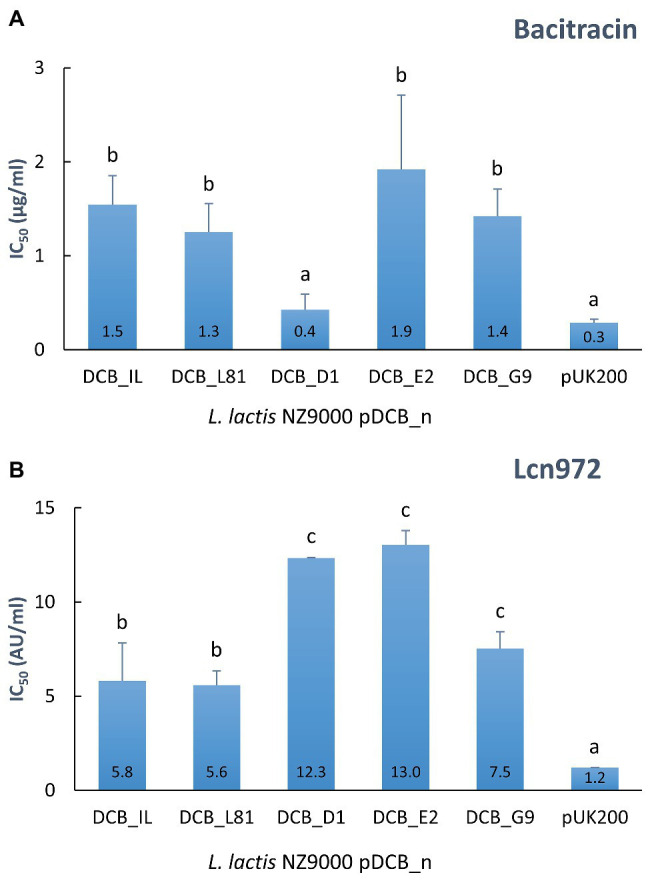
Resistance to bacitracin **(A)** and Lcn972 **(B)** of *L. lactis* NZ9000 clones expressing the different versions of *ysaDCB* genes. Calculated IC_50_ values are displayed inside the bars. Average and SD from, at least, two independent IC_50_ determinations are shown. Values with different letters are significantly different (*p* < 0.05).

In order to confirm the suspected role of the YsaDCB transporter in bacitracin and Lcn972 resistance and assess the impact of the *ysaB* mutations, the WT and mutated *ysaB* genes were cloned under the inducible nisin promoter (plasmids pDCB_n) and expressed in the *L. lactis* NZ9000 background (*ysaB* deficient). In this way, the level of protection provided by the ABC transporter itself and its mutated versions could be assessed and compared within the same genetic background, independently of their own regulation and the other mutations present in the Lcn972R mutants. The transporter genes of *L. lactis* IL1403 were also cloned (pDCB_IL) because they have been previously linked to nisin resistance (Kramer et al., 2006). Dose-response curves showed that expression of the WT genes *ysaDCB* of *L. lactis* L81 and *L. lactis* IL1403 conferred a 5-fold increase in the bacitracin and Lcn972 concentration required to inhibit growth by 50% (IC_50_), as compared to the control with the empty vector pUK200 (Figure 3). Hence, the role of YsaDCB in resistance of *L. lactis* to both AMPs was confirmed.

In the case of the mutated alleles of *ysaB*, represented by the *ysaDCB* operons from L81-D1, L81-E2, and L62-G9 (the WT *ysaDCB* from *L. lactis* L62 is identical to L81), contribution to resistance varied depending on the AMP (Figure 3). In line with the MICs reported in Table 2, the double *ysaB* mutant transporter pDCB_D1 failed to protect against bacitracin as judged by the low IC_50_ values, similar to those with the control *L. lactis* pUK200. On the contrary, the transporters in pDCB_E2 and pDCB_G9 provided similar levels of protection as the WT transporter. As for resistance to Lcn972, the scenario was different. Regardless of the *ysaB* allele, all the transporters boosted protection against Lcn972 over the WT genes (*p* < 0.05; Figure 3 IC_50_), with pDCB_G9 being less effective than pDCB_D1 and pDCB_E2 (*p* < 0.05). These results suggest that the mechanisms whereby YsaDCB protects *L. lactis* against bacitracin and Lcn972 might differ.

### *ysaB* Mutations Trigger the P*_ysaD_* Promoter in the Absence of Bacitracin

A mutagenesis study of the BceSR/BceAB resistance module has shown that single amino acid substitutions in the *B. subtilis* permease BceB may have different consequences in either signaling or resistance (Kallenberg et al., 2013). Moreover, the authors observed that those mutations that primarily affect the activity of the transporter tend to cluster in the C-terminal of the permease, which is the case with the *ysaB* mutations identified in the Lcn972R *L. lactis* mutants (see Table 1). Therefore, we next investigated if the mutations in *ysaB*, besides affecting resistance, could also alter onward signal transmission through the TCS-G and lead to induction of the *ysaDCB* operon upon detection of the stress. With this aim, bacitracin was chosen to measure P*_ysaD_* promoter activity due to the different resistance levels provided by the *ysaB* alleles (see Figure 3A). We first confirmed that P_ysaD_ was silent (OFF-state) in *L. lactis* NZ9000 lacking *ysaB*, even in the presence of bacitracin. As shown in Supplementary Figure S1B, while mCherry fluorescence in *L. lactis* IL1403/pRCR_P*_ysaD_*::*mrfp* increased proportionally to increasing bacitracin concentration, there was no response in *L. lactis* NZ9000/pRCR_P*_ysaD_*::*mrfp*. Therefore, *L. lactis* NZ9000 was deemed as a suitable host to study the signaling ability of YsaDCB and the existence of any other mechanisms that could induce P*_ysaD_* was ruled out.

**Figure 4:**
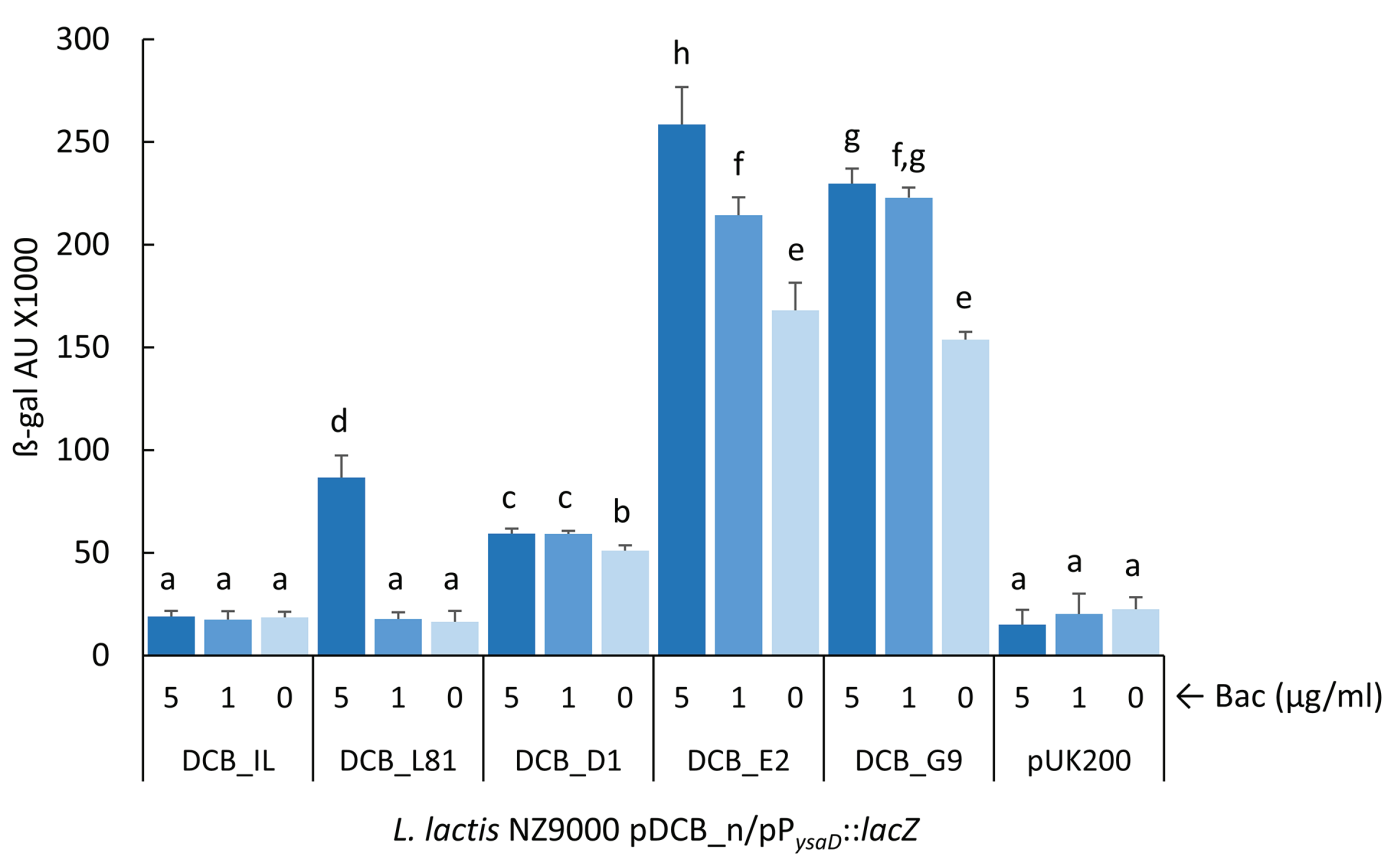
Induction of P*_ysaD_* by bacitracin. Exponentially growing *L. lactis* NZ9000 clones expressing the different versions of *ysaDCB* genes (pDCB_n) and carrying the reporter plasmid pP*_ysaD_*::*lacZ* were challenged with varying concentrations of bacitracin (Bac) for 30 min. Average and SD from three independent experiments are shown. Values with different letters are significantly different (*p* < 0.05).

To quantify the activity of the P_ysaD_ promoter in the *L. lactis* NZ9000 pDCB_n clones expressing the different *ysaB* alleles, the reporter plasmid pP*_ysaD_*::*lacZ* was constructed in which a Tet resistance marker is present to help keeping both pDCB_n and reporter plasmids in *L. lactis* NZ9000. Although the β-galactosidase assay was not sensitive enough to detect activation of P_ysaD_ in the presence of pDCB_IL, the promoter was responsive to bacitracin when the *L. lactis* L81 *ysa* genes (pDCB_L81) were expressed (Figure 4). Hence, it was possible to compare signaling driven by the different *ysaB* alleles. Under non-inducing conditions, *L. lactis* NZ9000 expressing the mutated *ysaB* transporters exhibited a higher P_ysaD_ basal activity than that achieved with the WT transporter YsaDCB_L81 (*p* < 0.05). This basal activity was remarkably high in the presence of YsaDCB_E2 and YsaDCB_G9 transporters (Figure 4). Besides this apparent deregulation, the P_ysaD_ promoter was still induced by bacitracin, although the level of response was clearly diminished in relation to the WT transporter. In this latter case, β-galactosidase activity rose by 5-fold after the challenge with bacitracin at 5 μg/ml, whereas it only increased 1.2-fold in the presence of YsaDCB_D1 and 1.5-fold and in the presence of the other two mutated transporters.

### The Secreted Peptide YsaD Seems to Modulate the Activity of the YsaDCB/TCS-G Module

The fact that *ysaD* forms an operon with *ysaCB* and that it is conserved within *L. lactis* raised the question whether this secreted peptide was somehow required for proper functioning of the YsaDCB/TCS-G module. Bearing in mind the dual role of the transporters in resistance and signal relay, we initially determined the IC_50_ values of bacitracin and Lcn972 for *L. lactis* NZ9000 expressing *ysaCB* from *L. lactis* IL1403 (pCB_IL) or from *L. lactis* L81 (pCB_L81) and compared to those expressing the whole cluster *ysaDCB* (Table 3). YsaD appears to be dispensable for resistance. All the clones were more resistant to bacitracin and Lcn972, compared to the control with the empty vector, and showed comparable bacitracin and Lcn972 IC_50_ values regardless the presence of *ysaD*. Only *L. lactis* NZ9000 pCB_L81 exhibited a 2-fold increase in the bacitracin IC_50_, compared to pDCB_L81, suggesting a possible but marginal role of *ysaD* in resistance.

**Table 3:** IC_50_ values*^a^* for *L. lactis* NZ9000 expressing *ysaCB*.

*L. lactis* NZ9000	Bacitracin (μg/ml)	Lcn972 (AU/ml)
pDCB_L81*^b^*	1.25 ± 0.30	5.58 ± 075
pCB_L81	2.41 ± 0.14^*^	6.63 ± 0.13
pDCB_IL*^b^*	1.55 ± 0.31	5.81 ± 2.02
pCB_IL	1.52 ± 0.23	4.45 ± 0.89
pUK200*^b^*	0.29 ± 0.01	1.21 ± 0.01

a Average ± standard deviation of, at least, two independent biological replicates are shown.

b IC_50_ values are also shown in Figure 3 but are included here for comparison.

* *p* < 0.05, significant difference between pDCB_L81 vs. pCB_L81. For all clones, bacitracin and Lcn972 IC_50_ are increased in comparison with *L. lactis* NZ9000 with the empty vector pUK200.

**Figure 5:**
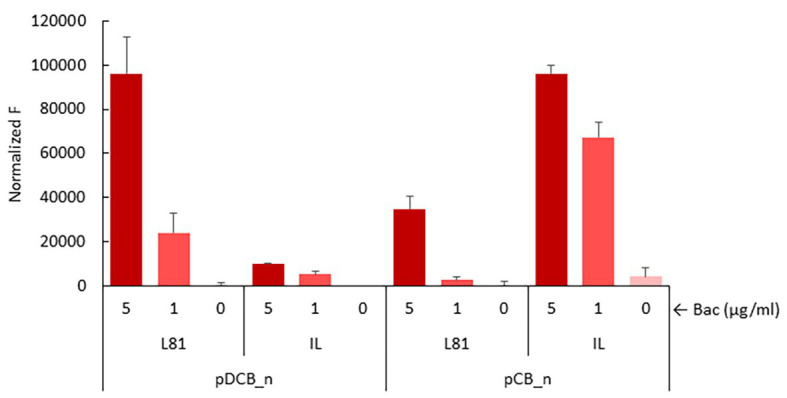
Induction of P*_ysaD_* in the presence of pDCB_n and pCB_n. Exponentially growing *L. lactis* NZ9000 clones expressing the full *ysaDCB* (DCB_n) operon or *ysaCB* (CB_n) lacking *ysaD* from *L. lactis* L81 (L81) or *L. lactis* IL1403 (IL) and the reporter plasmid pIL_P*_ysaD_*::*mrfp* were challenged with bacitracin at 5 and 1 μg/ml for 30 min before mCherry fluorescence (F) was measured.

We also measured P*_ysaD_* activity in the absence of *ysaD* to assess if YsaD could interfere with signal relay. Due to the low sensitivity of the *lacZ* reporter, unable to reveal induction of the P*_ysaD_* promoter by the *L. lactis* IL1403 YsaDCB transporter, a more sensitive reporter plasmid pIL_P*_ysaD_*::*mrfp*, based on the mCherry fluorescent protein, was used. This reporter was transferred to *L. lactis* NZ9000 pDCB_n and pCB_n clones. As shown in Figure 5, induction of P*_ysaD_* by bacitracin in the presence of pDCB_IL could be demonstrated, albeit the response was roughly 10-fold lower than with pDCB_L81 (Figure 5). When the activity of P*_ysaD_* was compared in the absence and presence of *ysaD* after the addition of bacitracin, the results were different depending on the transporter. The P*_ysaD_* promoter was turned down in *L. lactis* NZ9000/pCB_L81, while it was strongly induced in *L. lactis* NZ9000/pCB_IL (Figure 5). This apparently incongruent behavior must rely on subtle differences within the L81 and IL1403 ABC transporters that could be important for the interaction with the *L. lactis* NZ9000 TCS-G (see section Discussion). Nonetheless, YsaD appears to modulate the activity of the YsaDCB/TCS-G module by altering signal relay. Finally, it is worth noting that attempts to show induction of P*_ysaD_* by Lcn972 failed. All the reporter strains with either pDCB_n or pCB_n were exposed to Lcn972 at 80, 40, and 20 AU/ml, but fluorescence was never detected (data not shown).

## Discussion

The *L. lactis* YsaDCB ABC transporter is proposed to form a functional AMP detoxification module with the adjacent TCS-G, based on the genetic organization and the domain architecture of both the permease YsaB and the intramembrane-sensing histidine kinase KinG, two landmarks shared among AMP resistance modules in Firmicutes (Dintner et al., 2011; Gebhard, 2012). Accordingly, we have shown that increased expression of *ysaDCB* confers resistance against bacitracin and Lcn972 in *L. lactis* and that the module does not respond to bacitracin in the absence of a functional ABC transporter.

Previous studies have also linked this ABC transporter to nisin resistance in *L. lactis* (Kramer et al., 2006). Thereby, YsaDCB/TCS-G is able to provide resistance to cationic AMPs that differ in their mode of action. This is similar to other modules such as the staphylococcal GraXSR/VraFG and BraRS/BraDE reviewed by Kawada-Matsuo et al. (2011), whereas in *B. subtilis* the different Bce-like modules seem to be more specialized for bacitracin (BceSR/BceAB), nisin and other lipid II-binding lantibiotics (PsdRS/PsdAB) or cationic peptides such as the human defensin LL-37 (YxdJK/YxdLM-YxeA; Staron et al., 2011). The reason likely relies on the scope of the genes which are regulated by these resistance modules. Some modules only regulate the ABC transporter genes (e.g., BceSR/BceAB), while others (e.g., GraXSR/VraFG) include genes such as the *dlt* operon and *mprf* involved in d-alanylation of lipoteichoic acids and lysylination of phosphatidylglycerol, respectively, that contribute to AMP resistance by decreasing the net negative charge of the bacterial surface. BraRS/BraDE and VirSR/VirAB also regulate the expression of other ABC transporters such as VraDEH in *S. aureus* and AnrAB in *Listeria monocytogenes*, respectively. These transporters likely function as a “hydrophobic vacuum cleaner,” removing the AMPs out of their site of action (Collins et al., 2010; Hiron et al., 2011).

Our results have demonstrated that point mutations in *ysaB* activate the YsaDCB/TCS-G module. P*_ysaD_* is active in the absence of bacitracin, when the mutated *ysaB* alleles are expressed in *L. lactis* NZ9000 and not in the case of the WT transporters. Gain-of-function mutations in the Bra(Nsa)SR/BraDE module have been mapped on *braS* or *braR* or on the TCS promoter in nisin-resistant *S. aureus* (Randall et al., 2018; Arii et al., 2019), but there are also examples in which mutations in the permease gene enhance resistance by inducing the expression of the ABC-transporter genes (Becker et al., 2009). In view of the current flux sensing model (Fritz et al., 2015), one possible explanation could be that the permease mutations reduce the activity of the YsaDCB transporter. This scenario would impose more pressure onto the system to increase the rate of *de novo* transporter synthesis and get more transporter molecules to relieve the stress. However, it is difficult to explain the constitutive expression in the absence of the AMP and the loss of responsiveness to AMP concentration, as observed with the mutated YsaDCB transporters. Moreover, since flux sensing relies on ATP hydrolysis, it would be clearly deleterious for the cells. Alternatively, the mutations in the YsaB permease could modify protein-protein interactions within the module, triggering activation of the histidine kinase, irrespectively of the activity of the YsaDCB transporter. Recently, it has been proposed that the ABC transporter BraDE, beyond its role in sensing and activation of BraSR regulon, also enables onward signal transduction through the histidine kinase BraS in some way, which is independent of ATP consumption (Randall et al., 2018). This role was proposed after observing that the nisin-resistant phenotype of a gain-of-function *braS* mutant lacking *braDE* could be complemented with an ATP-deficient *braDE* copy. In this scenario, it is conceivable that mutations in the permease could prompt activation of the TCS. However, further biochemical evidence is required to prove if this is the case of the *ysaB* mutations.

Increased expression of *ysaDCB*-D1 was not enough to confer resistance to bacitracin, whereas protection against Lcn972 was enhanced compared to the WT YsaDCB transporter. This takes us to speculate that YsaDCB may protect *L. lactis* by two different means. For bacitracin, it has been proposed that the *B. subtilis* BceAB transporter binds to the complex bacitracin-undecaprenyl pyrophosphate and releases the antibiotic from it, freeing the cell wall precursor to proceed with cell wall biosynthesis (Kobras et al., 2020). The double amino acid substitution F_577_S/P_605_T in YsaB-D1 could make the ABC transporter less efficient for bacitracin release, and thus unable to protect against bacitracin. Kobras et al. (2020) also hypothesize that a similar target protection mechanism should also work for lipid II binding peptides such as mesarcidin and actagardine. However, YsaDCB-D1 is as effective as YsaDCB-E2 against Lcn972. Since YsaDCB-D1 is still competent to trigger activation of the module, we speculate that downstream activities (i.e., other functions regulated by the YsaDCB/TCS-G module) are responsible for resistance to Lcn972. In support of this hypothesis, motif-based searches, using the consensus binding sequence for BceR-like response regulators proposed by Dintner et al. (2011), detected a putative BceR-binding box in the promoter of the *dlt* genes in *L. lactis*. Moreover, d-alanylation of lipoteichoic acids is known to constitute an Lcn972 resistance factor (Roces et al., 2012).

The *L. lactis* BceAB-like transporter is equipped with an additional protein YsaD whose structural gene is co-transcribed with *ysaCB*. According to BlastP searches, YsaD belongs to the family TIGR01655 of small uncharacterized proteins with N-terminal signal sequences, conserved in Gram-positive organisms. These properties are shared with YxeA, in the *B. subtilis* module YxdJK/YxdLM-YxeA (Joseph et al., 2004), but its putative function has not been addressed so far. Our results suggest that these proteins may play an accessory role, modulating the activity of the transporter and/or governing signal transduction through protein-protein interactions within the module. Despite having an identical YsaD, removal of this protein had two opposite effects: a drop of signaling in the case of the transporter YsaDCB-L81 and boosting the otherwise poor ability of the *L. lactis* IL1403 transporter to activate *L. lactis* NZ9000 TCS-G (see Figure 5). Being aware that our experimental setup is not the best scenario to interrogate for fine tuning or regulatory feedback, simply because it relies on the formation of a heterologous ABC/TCS module, this discrepancy could be explained when specific YsaD-YsaB or YsaD-YsaB-KinG interactions regulate transport activity and that such interactions differ within these two transporters. Both YsaDCB-L81 and YsaDCB-IL are highly homologous with only one non-conservative amino acid substitution in the ATP-binding protein YsaC (E_139_ in L81 and K_139_ in IL1403), outside the conserved protein domains and unlikely to interfere with transport activity. The other one is in the YsaB permease (K_621_ in L81 and E_621_ in IL1403). This position is predicted to be located at the external linker between the last two TMHs IX and X. From our results and others (Kallenberg et al., 2013), it is known that single amino acid substitutions in the permease, namely, in the C-terminus, may severely alter signal transduction. Consistent with this, there is a 10-fold difference in the ability of the IL1403 and L81 transporters to trigger the P*_ysaD_* promoter in response to bacitracin. ABC connectors or accessory proteins such as the cytosolic GraX and the small membrane protein VraH have already been described (Falord et al., 2012; Popella et al., 2016). Whether the same applies for a soluble and likely secreted peptide remains to be investigated. It is worth mentioning that in *L. lactis* there is an additional gene, *ysaA*, which is co-transcribed with the TCS-G gene and specifies for a transmembrane protein. YsaA, as yet unique to *L. lactis*, is likely to play a role in the transduction pathway too.

There was another result that will require further attention. Under the experimental conditions used in this work, we were unable to detect activation of P*_ysaD_* by Lcn972, which is known to bind to lipid II as many other Bce-like inducers. In a way, this was unexpected given that the gain-of-function mutations in *ysaB* were selected under the stress imposed by this bacteriocin (López-González et al., 2018). However, YsaDCB/TCS-G is likely regulating the *dlt* operon, which should protect against Lcn972 and, thereby, growth of gain-of-function mutants would be expedited. At this stage, it is difficult to propose a reasonable explanation for the lack of induction by Lcn972. We cannot disregard a host effect, i.e., that Lcn972 is unable to trigger the response in *L. lactis* NZ9000 but in other strain backgrounds, as recently observed with VirAB/VirRS in *L. monocytogenes* (Grubaugh et al., 2018; Jiang et al., 2019).

In conclusion, we have shown that YsaDCB/TCS-G participates in AMP resistance in *L. lactis* anticipating alternative mechanisms of resistance against different AMPs. Specific mutations which are easily selected under cell envelope stress have been shown to activate the module constitutively. Moreover, YsaDCB/TCS-G seems to be unique as to the intricate regulatory circuits due to the presence of additional proteins that modulate its activity, thereby warranting further research to understand these important resistance factors and their contribution to antimicrobial resistance. While for a nonpathogenic bacterium such as *L. lactis* antibiotic resistance may not be an issue of concern so far, this study opens new avenues for developing biotechnologically proficient strains able to withstand better the presence of bacteriocins or other cationic antimicrobials as lysozyme, currently in use as preservatives in food.

## Data Availability Statement

The original contributions presented in the study are included in the article/Supplementary Material, and further inquiries can be directed to the corresponding author.

## Author Contributions

AR and BM conceived and designed the study. AC, ML-G, SE, and BM performed the experiments. AN and TJ contributed with strains and materials. AR and BM wrote the draft manuscript. All authors participated in the interpretation of the results and read and approved the manuscript.

### Conflict of Interest

AN and TJ are employees of Chr. Hansen A/S.

The remaining authors declare that the research was conducted in the absence of any commercial or financial relationships that could be construed as a potential conflict of interest.

## Footnotes

^1^https://embnet.vital-it.ch/software/TMPRED_form.html

^2^https://www.psort.org/psortb/

^3^https://blast.ncbi.nlm.nih.gov/Blast.cgi

## References

[ref1] Arii K. Kawada-Matsuo M. Oogai Y. Noguchi K. Komatsuzawa H. (2019). Single mutations in BraRS confer high resistance against nisin a in *Staphylococcus aureus*. Microbiologyopen 8:e791. 10.1002/mbo3.791, PMID: 30656859 PMC6854852

[ref2] Becker P. Hakenbeck R. Henrich B. (2009). An ABC transporter of *Streptococcus pneumoniae* involved in susceptibility to vancoresmycin and bacitracin. *Antimicrob. Agents Chemother*. 53, 2034–2041. 10.1128/AAC.01485-08, PMID: 19273682 PMC2681518

[ref3] Bernard R. Guiseppi A. Chippaux M. Foglino M. Denizot F. (2007). Resistance to bacitracin in *Bacillus subtilis*: unexpected requirement of the BceAB ABC transporter in the control of expression of its own structural genes. J. Bacteriol. 189, 8636–8642. 10.1128/JB.01132-07, PMID: 17905982 PMC2168949

[ref4] Bolotin A. Wincker P. Mauger S. Jaillon O. Malarme K. Weissenbach J.. (2001). The complete genome sequence of the lactic acid bacterium *Lactococcus lactis* ssp. *lactis* IL1403. Genome Res. 11, 731–753. 10.1101/gr.GR-1697R, PMID: 11337471 PMC311110

[ref5] Burgess C. O’Connell-Motherway M. Sybesma W. Hugenholtz J. van Sinderen D. (2004). Riboflavin production in *Lactococcus lactis*: potential for in situ production of vitamin-enriched foods. Appl. Environ. Microbiol. 70, 5769–5777. 10.1128/AEM.70.10.5769-5777.2004, PMID: 15466513 PMC522069

[ref6] Campelo A. B. Rodriguez A. Martinez B. (2010). Use of green fluorescent protein to monitor cell envelope stress in *Lactococcus lactis*. Appl. Environ. Microbiol. 76, 978–981. 10.1128/AEM.02177-09, PMID: 19948854 PMC2813010

[ref7] Collins B. Curtis N. Cotter P. D. Hill C. Ross R. P. (2010). The ABC transporter AnrAB contributes to the innate resistance of *Listeria monocytogenes* to nisin, bacitracin, and various beta-lactam antibiotics. Antimicrob. Agents Chemother. 54, 4416–4423. 10.1128/AAC.00503-10, PMID: 20643901 PMC2944581

[ref8] Dintner S. Heermann R. Fang C. Jung K. Gebhard S. (2014). A sensory complex consisting of an ATP-binding cassette transporter and a two-component regulatory system controls bacitracin resistance in *Bacillus subtilis*. J. Biol. Chem. 289, 27899–27910. 10.1074/jbc.M114.596221, PMID: 25118291 PMC4183823

[ref9] Dintner S. Staron A. Berchtold E. Petri T. Mascher T. Gebhard S. (2011). Coevolution of ABC transporters and two-component regulatory systems as resistance modules against antimicrobial peptides in Firmicutes bacteria. J. Bacteriol. 193, 3851–3862. 10.1128/JB.05175-11, PMID: 21665979 PMC3147537

[ref10] Du D. Wang-Kan X. Neuberger A. van Veen H. W. Pos K. M. Piddock L. J. V.. (2018). Multidrug efflux pumps: structure, function and regulation. Nat. Rev. Microbiol. 16, 523–539. 10.1038/s41579-018-0048-6, PMID: 30002505

[ref11] Falord M. Karimova G. Hiron A. Msadek T. (2012). GraXSR proteins interact with the VraFG ABC transporter to form a five-component system required for cationic antimicrobial peptide sensing and resistance in *Staphylococcus aureus*. Antimicrob. Agents Chemother. 56, 1047–1058. 10.1128/AAC.05054-11, PMID: 22123691 PMC3264281

[ref12] Fritz G. Dintner S. Treichel N. S. Radeck J. Gerland U. Mascher T.. (2015). A new way of sensing: need-based activation of antibiotic resistance by a flux-sensing mechanism. mBio 6:e00975. 10.1128/mBio.00975-15, PMID: 26199330 PMC4513084

[ref13] Garay-Novillo J. N. Garcia-Morena D. Ruiz-Maso J. A. Barra J. L. Del Solar G. (2019). Combining modules for versatile and optimal labeling of lactic acid bacteria: two pMV158-family promiscuous replicons, a pneumococcal system for constitutive or inducible gene expression, and two fluorescent proteins. Front. Microbiol. 10:1431. 10.3389/fmicb.2019.01431, PMID: 31297101 PMC6607859

[ref14] Gebhard S. (2012). ABC transporters of antimicrobial peptides in Firmicutes bacteria—phylogeny, function and regulation. Mol. Microbiol. 86, 1295–1317. 10.1111/mmi.12078, PMID: 23106164

[ref15] Grubaugh D. Regeimbal J. M. Ghosh P. Zhou Y. Lauer P. Dubensky T. W. Jr.. (2018). The VirAB ABC transporter is required for VirR regulation of *Listeria monocytogenes* virulence and resistance to nisin. Infect. Immun. 86, e00901–e00917. 10.1128/IAI.00901-17, PMID: 29263107 PMC5820956

[ref16] Hiron A. Falord M. Valle J. Debarbouille M. Msadek T. (2011). Bacitracin and nisin resistance in *Staphylococcus aureus*: a novel pathway involving the BraS/BraR two-component system (SA2417/SA2418) and both the BraD/BraE and VraD/VraE ABC transporters. Mol. Microbiol. 81, 602–622. 10.1111/j.1365-2958.2011.07735.x, PMID: 21696458

[ref17] Jiang X. Geng Y. Ren S. Yu T. Li Y. Liu G.. (2019). The VirAB-VirSR-AnrAB multicomponent system is involved in resistance of *Listeria monocytogenes* EGD-e to cephalosporins, bacitracin, nisin, benzalkonium chloride, and ethidium bromide. Appl. Environ. Microbiol. 85, e01470–e01519. 10.1128/AEM.01470-19, PMID: 31399408 PMC6805085

[ref18] Joseph P. Guiseppi A. Sorokin A. Denizot F. (2004). Characterization of the *Bacillus subtilis* YxdJ response regulator as the inducer of expression for the cognate ABC transporter YxdLM. Microbiology 150, 2609–2617. 10.1099/mic.0.27155-0, PMID: 15289557

[ref19] Kallenberg F. Dintner S. Schmitz R. Gebhard S. (2013). Identification of regions important for resistance and signalling within the antimicrobial peptide transporter BceAB of *Bacillus subtilis*. J. Bacteriol. 195, 3287–3297. 10.1128/JB.00419-13, PMID: 23687272 PMC3697649

[ref20] Kawada-Matsuo M. Yoshida Y. Nakamura N. Komatsuzawa H. (2011). Role of two-component systems in the resistance of *Staphylococcus aureus* to antibacterial agents. Virulence 2, 427–430. 10.4161/viru.2.5.17711 21921684

[ref21] Khosa S. AlKhatib Z. Smits S. H. (2013). NSR from *Streptococcus agalactiae* confers resistance against nisin and is encoded by a conserved nsr operon. Biol. Chem. 394, 1543–1549. 10.1515/hsz-2013-0167, PMID: 23893686

[ref22] Kobras C. M. Piepenbreier H. Emenegger J. Sim A. Fritz G. Gebhard S. (2020). BceAB-type antibiotic resistance transporters appear to act by target protection of cell wall synthesis. Antimicrob. Agents Chemother. 64, e02241–e02319. 10.1128/AAC.02241-19, PMID: 31871088 PMC7038271

[ref23] Kramer N. E. van Hijum S. A. Knol J. Kok J. Kuipers O. P. (2006). Transcriptome analysis reveals mechanisms by which *Lactococcus lactis* acquires nisin resistance. Antimicrob. Agents Chemother. 50, 1753–1761. 10.1128/AAC.50.5.1753-1761.2006, PMID: 16641446 PMC1472215

[ref24] Kuipers O. P. de Ruyter P. G. G. A. Kleerebezem M. de Vos W. M. (1998). Quorum sensing-controlled gene expression in lactic acid bacteria. J. Biotechnol. 64, 15–21. 10.1016/S0168-1656(98)00100-X

[ref25] Livak K. J. Schmittgen T. D. (2001). Analysis of relative gene expression data using real-time quantitative PCR and the 2^−ΔΔCT^ method. Methods 25, 402–408. 10.1006/meth.2001.1262, PMID: 11846609

[ref26] López-González M. J. Escobedo S. Rodríguez A. Neves A. R. Janzen T. Martínez B. (2018). Adaptive evolution of industrial *Lactococcus lactis* under cell envelope stress provides phenotypic diversity. Front. Microbiol. 9:2654. 10.3389/fmicb.2018.02654 30455679 PMC6230721

[ref27] Martínez B. Böttiger T. Schneider T. Rodríguez A. Sahl H. G. Wiedemann I. (2008). Specific interaction of the unmodified bacteriocin Lactococcin 972 with the cell wall precursor lipid II. Appl. Environ. Microbiol. 74, 4666–4670. 10.1128/AEM.00092-08, PMID: 18539790 PMC2519333

[ref28] Martínez B. Zomer A. L. Rodríguez A. Kok J. Kuipers O. P. (2007). Cell envelope stress induced by the bacteriocin Lcn972 is sensed by the lactococcal two-component system CesSR. Mol. Microbiol. 64, 473–486. 10.1111/j.1365-2958.2007.05668.x, PMID: 17493129

[ref29] Mohedano M. L. García-Cayuela T. Perez-Ramos A. Gaiser R. A. Requena T. López P. (2015). Construction and validation of a mCherry protein vector for promoter analysis in *Lactobacillus acidophilus*. J. Ind. Microbiol. Biotechnol. 42, 247–253. 10.1007/s10295-014-1567-4, PMID: 25533634

[ref30] Piepenbreier H. Fritz G. Gebhard S. (2017). Transporters as information processors in bacterial signalling pathways. Mol. Microbiol. 104, 1–15. 10.1111/mmi.13633, PMID: 28152228

[ref31] Popella P. Krauss S. Ebner P. Nega M. Deibert J. Gotz F. (2016). VraH is the third component of the *Staphylococcus aureus* VraDEH system involved in Gallidermin and Daptomycin resistance and pathogenicity. Antimicrob. Agents Chemother. 60, 2391–2401. 10.1128/AAC.02865-15, PMID: 26856834 PMC4808217

[ref32] Randall C. P. Gupta A. Utley-Drew B. Lee S. Y. Morrison-Williams G. O’Neill A. J. (2018). Acquired nisin resistance in *Staphylococcus aureus* involves constitutive activation of an intrinsic peptide antibiotic detoxification module. mSphere 3, e00633–e00718. 10.1128/mSphereDirect.00633-18, PMID: 30541781 PMC6291627

[ref33] Reiners J. Lagedroste M. Ehlen K. Leusch S. Zaschke-Kriesche J. Smits S. H. J. (2017). The N-terminal region of nisin is important for the BceAB-type ABC transporter NsrFP from *Streptococcus agalactiae* COH1. Front. Microbiol. 8:1643. 10.3389/fmicb.2017.01643, PMID: 28912758 PMC5583591

[ref34] Revilla-Guarinos A. Gebhard S. Mascher T. Zuniga M. (2014). Defence against antimicrobial peptides: different strategies in Firmicutes. Environ. Microbiol. 16, 1225–1237. 10.1111/1462-2920.12400, PMID: 24548478

[ref35] Roces C. Courtin P. Kulakauskas S. Rodríguez A. Chapot-Chartier M. P. Martínez B. (2012). Isolation of *Lactococcus lactis* mutants simultaneously resistant to the cell wall-active bacteriocin Lcn972, lysozyme, nisin and bacteriophage c2. Appl. Environ. Microbiol. 78, 4157–4163. 10.1128/AEM.00795-12, PMID: 22504807 PMC3370530

[ref36] Staron A. Finkeisen D. E. Mascher T. (2011). Peptide antibiotic sensing and detoxification modules of *Bacillus subtilis*. Antimicrob. Agents Chemother. 55, 515–525. 10.1128/AAC.00352-10, PMID: 21078927 PMC3028804

[ref37] Wegmann U. Klein J. R. Drumm I. Kuipers O. P. Henrich B. (1999). Introduction of peptidase genes from *Lactobacillus delbrueckii* subsp. *lactis* into *Lactococcus lactis* and controlled expression. Appl. Environ. Microbiol. 65, 4729–4733. 10.1128/AEM.65.11.4729-4733.1999, PMID: 10543778 PMC91636

