# Supplementary material for: Mutations Selected After Exposure to Bacteriocin Lcn972 Activate a Bce-Like Bacitracin Resistance Module in Lactococcus lactis
Source: Front Microbiol. 2020 Aug 13;11:1805. doi: 10.3389/fmicb.2020.01805 (PMC7438565; doi:10.3389/fmicb.2020.01805)
Supplement: Supplementary file 3 [file Data_Sheet_1.docx]

Supplementary Material

**Mutations selected after exposure to bacteriocin Lcn972 activate a Bce-like bacitracin resistance module in *Lactococcus lactis***

**Ana Belén Campelo^1^, Mª Jesús López-González^1,2^, Susana Escobedo^1,2^, Thomas Janzen^3^, Ana Rute Neves^3^, Ana Rodríguez^1,2^, Beatriz Martínez^1,2,*^**

^1^ DairySafe group. Instituto de Productos Lácteos de Asturias (IPLA), Technology and Biotecnology of Dairy Products Department, Consejo Superior de Investigaciones Científicas (CSIC). Villaviciosa, Asturias, Spain.

^2^ Instituto de Investigación Sanitaria del Principado de Asturias (ISPA), Oviedo, Asturias, Spain.

^3^ Chr Hansen A/S. Hørsholm, Denmark.

*** Correspondence:**

Beatriz Martínez

bmf1@ipla.csic.es


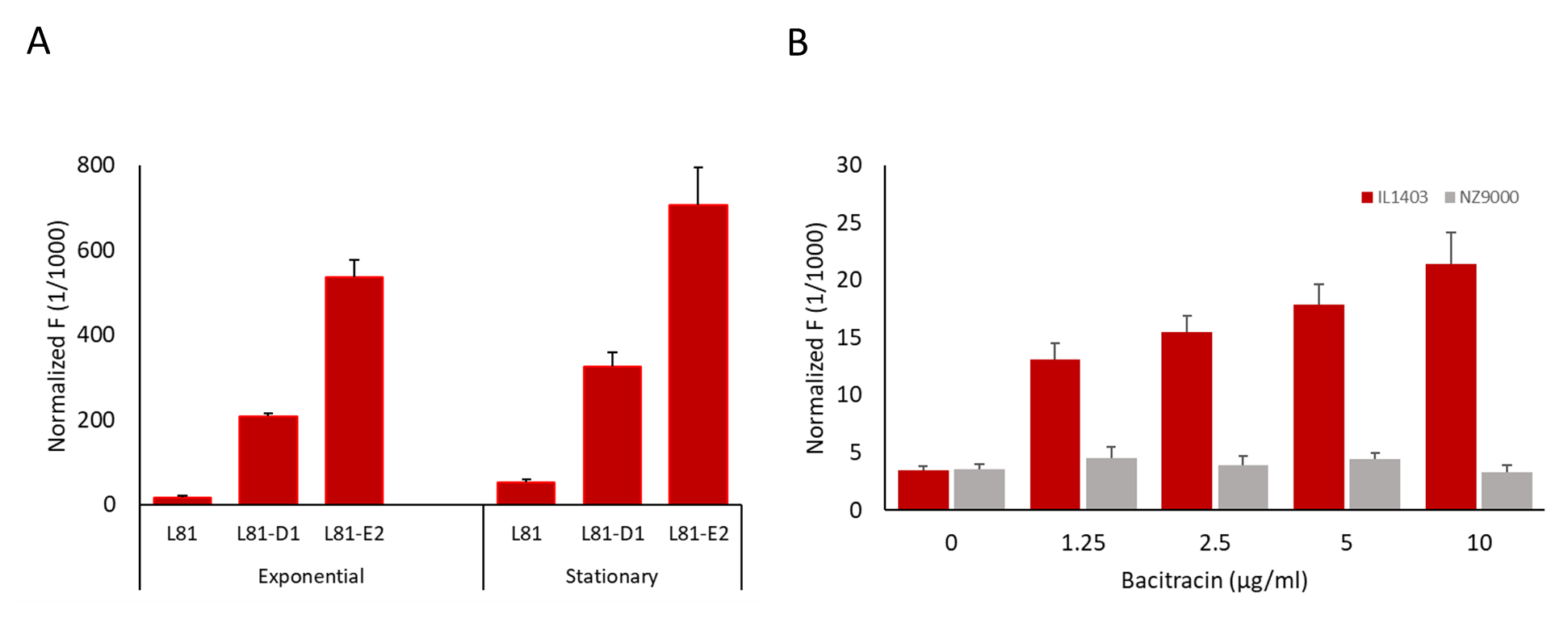


**Supplementary Figure 1. Activity of the P*_ysaD_* promoter in *L. lactis* determined by the reporter plasmid pPRC_P*_ysaD_*::*mrfp*.** Detection of mCherry fluorescence (F) was recorded at 605 nm after excitation at a wavelength of 580 nm and normalized by the OD_600_ of the cell suspensions. A. Cells from *L. lactis* L81, L81-D1 and L81-E2 exponential and stationary phase cultures (only one biological replicate was analyzed, error bars are from technical replicates). B. *L. lactis* IL1403 (red bars) and NZ9000 (grey bars) after induction with bacitracin (X-axis). Average and standard deviation of three biological replicates is depicted.
